# Predicting one-year outcomes in first-episode psychosis

**DOI:** 10.1192/j.eurpsy.2021.65

**Published:** 2021-08-13

**Authors:** M. Lindgren, J. Keinänen, A. Kemppainen, B. Karpov, T. Kieseppä, J. Suvisaari

**Affiliations:** 1 Mental Health, Finnish Institute for Health and Welfare, Helsinki, Finland; 2 Department Of Psychiatry, University of Helsinki and Helsinki University Hospital, Helsinki, Finland; 3 Faculty Of Medicine And Health Technology, Tampere University, Tampere, Finland

**Keywords:** Psychotic disorders, remission, follow-up, outcome

## Abstract

The outcome of first-episode psychosis (FEP) varies and may be predicted by several baseline measures. In the Helsinki Early Psychosis Study, young adults with FEP (n=97) from the Helsinki area in Finland were broadly assessed as soon as possible after first psychiatric contact for psychosis. Age- and gender-matched population controls were also assessed (n=62). The participants were followed up via appointments and medical records. We present both published and unpublished results on predictors of 12-month clinical, functional, and metabolic outcomes. More severe cognitive deficits at the beginning of treatment predicted several outcomes such as occupational status and functional level – beyond baseline positive and affective symptom levels, but not when negative symptoms were accounted for. More severe baseline obsessive-compulsive symptoms were predictive of a lower rate of remission, whereas a higher level of anxiety symptoms predicted better functional outcome, when the severity of positive symptoms was adjusted for. Adverse childhood experiences measuring cumulating psychosocial stress did not predict occupational status or functional level when positive and negative symptoms and neurocognition were controlled for, whereas in controls having experienced school bullying was associated with lower functioning. Insulin resistance in early psychosis appeared as an early marker of increased vulnerability to weight gain and abdominal obesity in young adults with FEP. Further, increased waist circumference predicted worsening low-grade inflammation, increasing further the cardiovascular risk. In sum, we have found different types of prognostic markers in FEP. Identifying the individuals at risk of less favorable outcomes could affect treatment choices in FEP.

**Disclosure:**

No significant relationships.

